# Public health round-up 

**DOI:** 10.2471/BLT.18.010618

**Published:** 2018-06-01

**Preventing dengue through vaccination**

Only people who have already had dengue should be vaccinated with the vaccine, CYD-TDV (Dengvaxia®), according to a revised recommendation by the Strategic Advisory Group of Experts on Immunization (SAGE). The decision announced on 18 April, follows the disclosure of new long-term safety data on Dengvaxia® by its manufacturer, Sanofi Pasteur, in November 2017. Additional analyses revealed the need for further research into use of the vaccine in people who have not had a past dengue infection. For countries considering vaccination as part of their dengue programme, the SAGE recommended a pre-vaccination screening strategy to determine whether people have already had dengue, before vaccinating them with Dengvaxia®. The SAGE is the principal advisory group to the World Health Organization (WHO) for vaccines and immunization. This photograph is from a dengue immunization programme in Paraná state of Brazil. bit.ly/2KGyLah

**Figure:**
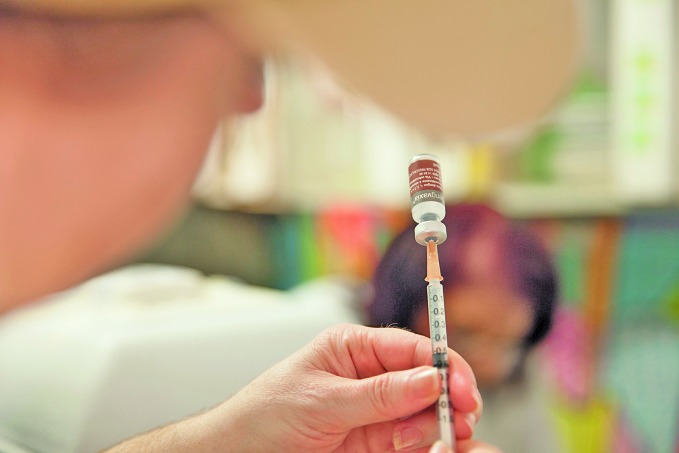
Sanofi Pasteur/Gabriel Lehto

## Cholera vaccination in Africa

Cholera vaccination campaigns targeting two million people in Zambia, Uganda, Malawi, South Sudan and Nigeria are set to be completed this month, in response to a recent spate of outbreaks. 

The campaigns are being implemented by health ministries and supported by the World Health Organization (WHO) and other partners from the Global Task Force on Cholera Control. 

The vaccines are being funded by Gavi, the Vaccine Alliance, and delivered from the global oral cholera vaccine stockpile. 

Between 1997 and 2012, some 1.5 million doses of cholera vaccines were used worldwide. In 2017, however, almost 11 million doses of the cholera vaccine were used in many countries including Bangladesh, Sierra Leone and Somalia. 

More than 15 million doses were approved for use worldwide in the first four months of this year alone.

The burden of cholera remains high in many African countries. As of 7 May, 12 countries or territories were reporting active cholera transmission in sub-Saharan Africa. 

Since January, WHO has been providing technical expertise and guidance, working closely with health ministries in the five countries, to plan and implement the oral cholera vaccination campaigns with different partners. 

Last year, WHO and other partners from the Global Task Force on Cholera Control launched a strategy to reduce cholera deaths globally by 90% by 2030. 

In addition to vaccination, the provision of basic water sanitation and hygiene in communities affected by cholera are vital for achieving this goal. 

bit.ly/2I1n5gv

## Draft guidelines 

WHO is reviewing comments received through a public consultation last month on its draft *Guidelines: Saturated fatty acid and trans-fatty acid intake for adults and children* and will revise the draft, if necessary. 

The draft guidelines recommend that adults and children keep the intake of saturated fatty acids to below 10% of their total energy intake, and of trans-fatty acids to below 1% of their total energy intake. The guidelines also suggest their replacement with polyunsaturated fatty acids. 

High intakes of saturated fatty acids and trans-fatty acids have been shown to increase the risk of cardiovascular disease and death. 

Cardiovascular diseases are the leading cause of death globally. 

Saturated fatty acids are found in animal products, such as butter, milk, meat, salmon and egg yolks, and some plant-derived products, such as chocolate, cocoa butter, coconut, and palm and palm kernel oils. 

Most dietary trans-fatty acids are produced industrially by the partial hydrogenation of vegetable and fish oils. Industrially-produced trans-fatty acids can be found in baked and fried foods, pre-packaged snacks and foods, and partially hydrogenated cooking oils and fats used at home, in restaurants, or by street vendors. 

The development of the guidelines is part of WHO’s efforts to update dietary goals for NCDs prevention. 

WHO has already released guidelines on recommended intakes of sodium, potassium and free sugars and is developing guidelines on the intake of total fat, polyunsaturated fatty acids, carbohydrates other than sugars (including starch quality, dietary fibre and fruits and vegetables), non-sugar sweeteners and dietary patterns. 

bit.ly/2jHSk5L

## Air pollution 

WHO estimates that some 7 million people die every year from exposure to fine airborne particles that are carried into the lungs and cardiovascular system, causing stroke, heart disease, lung cancer, respiratory conditions and other diseases. 

Air pollution levels remain dangerously high in many parts of the world, with 9 out of every 10 people breathing air containing high levels of pollutants, according to new data released by WHO last month. 

Ambient (outdoor) air pollution alone caused some 4.2 million deaths in 2016, while household air pollution generated from cooking with polluting fuels and technologies caused an estimated 3.8 million deaths in the same period. About 1 million of these deaths were caused by both indoor and outdoor pollution. 

Over the past 6 years, WHO estimates show ambient air pollution levels have remained high and relatively stable, with declining concentrations in some parts of Europe and the Americas. 

The highest ambient air pollution levels are in the Eastern Mediterranean Region and in South-East Asia, with annual mean levels often exceeding more than five times WHO limits, followed by low- and middle-income cities in Africa and the Western Pacific.

Data gaps still persist, particularly in WHO’s Africa Region and some parts of the the Western Pacific Region, although data coverage is generally improving. For the African Region, the database now contains particulate matter measurements for more than twice as many cities as previous versions, however, data was identified for only 8 of 47 countries in the region.

Europe has the highest number of cities reporting data. 

More than 4300 cities in 108 countries are now included in WHO’s ambient air quality database, making this the world’s most comprehensive database on ambient air pollution. Since 2016, more than 1000 additional cities have been added to WHO’s database. 

WHO will convene the first Global Conference on Air Pollution and Health from 30 October to 1 November, to bring governments and partners together in a global effort to improve air quality and combat climate change. 

bit.ly/2I1zfKn

**Cover photo**

This girl has just been vaccinated against yellow fever at the Clínica da Família Estácio de Sá in Rio de Janeiro. Brazil is facing an upsurge of the mosquito-borne disease with unusual patterns of spread, with more than 2000 cases since 2016.

**Figure:**
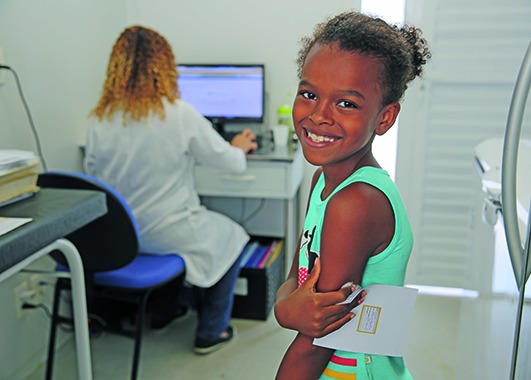
PAHO/WHO

## Listeriosis in South Africa

WHO urges governments worldwide to invest more in strengthening food safety systems while the response to the world’s largest known outbreak of listeriosis, a serious foodborne disease, continues in South Africa. 

A national multisectoral task force has been established in South Africa to coordinate the outbreak investigation and response. Last month, the outbreak was showing a downward trend after the food source – a processed meat product called polony – was identified and recalled in early March. The implicated product was also exported to some 15 countries in southern Africa. WHO, through the International Food Safety Authorities Network, facilitated the exchange of information on product distribution to enable these countries to take appropriate management measures, including local recalls and consumer education.

To prevent similar events and to ensure a safe food supply, WHO calls on governments to strengthen the different elements of national food safety systems, adopt international food standards developed by the Codex Alimentarius Commission, and strengthen foodborne disease surveillance, by implementing the recently published WHO guidance entitled *Strengthening surveillance of and response to foodborne diseases*. 

Countries are also urged to pay close attention to other common foodborne pathogens such as *Salmonella species*, *Campylobacter jejuni* and *Escherichia coli*. 

South Africa has been facing an outbreak of listeriosis since early 2017. 

Between 1 January 2017 and 24 April 2018, 1024 laboratory-confirmed listeriosis cases, including 200 deaths (out of 700 cases with known outcomes), were reported to the National Institute for Communicable Diseases from all the country’s provinces. The case fatality rate of 28.6% seen in this outbreak is similar to listeriosis outbreaks in other countries. 

Emergency response activities include surveillance (detection and investigation of cases), risk communication activities and food safety legislative review and reform.

bit.ly/2HZ31PP

## Managing epidemics

A new manual released last month provides essential guidance and information on how to respond effectively and rapidly at the start of an outbreak. 

The manual,**entitled* Managing epidemics, *is designed for WHO country representatives as well as government officials, nongovernmental organizations and other public health professionals. 

It focuses on 15 infectious diseases with the potential to become international threats: Ebola virus disease, Lassa fever, Crimean-Congo haemorrhagic fever, yellow fever, Zika virus, chikungunya, avian and other zoonotic influenza, seasonal influenza, pandemic influenza, Middle-East respiratory syndrome, cholera, monkeypox, plague, leptospirosis and meningococcal meningitis.

The manual was developed in parallel with the creation of OpenWHO (https://openwho.org), WHO’s new interactive, web-based, knowledge-transfer platform offering online courses on responding to health emergencies. 

bit.ly/2wjMalG

**Looking ahead**

**14 June – World Blood Donor Day**

**28 July – World Hepatitis Day**

**26 September – United Nations General Assembly high-level meeting on ending tuberculosis, New York**

**9 October ­– WHO 2018 Symposium on health financing for universal health coverage**

**30 October–1 November – WHO Global Conference on Air Pollution and Health, Geneva **

**1 December – World AIDS Day**

